# Breaking vaccination barriers among migrants? Human rights and crisis preparedness

**DOI:** 10.1093/medlaw/fwaf004

**Published:** 2025-02-07

**Authors:** Yana Litins’ka

**Affiliations:** Department of Law, Lund University, Lund 221 00, Sweden

**Keywords:** COVID-19, migrants, right to health, Sweden, temporary protection, vaccination

## Abstract

Vaccination hesitancy is one of the critical threats to public health. The coronavirus disease pandemic reconfirmed that certain groups of populations are more reluctant to vaccinate than others, particularly migrants. This article examines legal obligations related to protecting the right to health in addressing vaccination barriers among newly arrived adult migrants, taking Ukrainians granted temporary protection as an example. From human rights law requirements delineated by the United Nations and Council of Europe, it maps out a framework of vaccination-related obligations. Furthermore, the article tests the framework created in one national legal system—Sweden—to show where the gaps in transposing obligations into national law still exist. To deepen the analysis, the interview study with Ukrainian refugees in Sweden is presented, which allows reflection on what obligations have not reached their recipients and resulted in vaccination barriers. The article advocates for further specification of obligations related to vaccinations in both national and international laws for better crisis preparedness.

## I. INTRODUCTION

From 2020 to 2023, the world lived through the coronavirus disease (COVID-19) pandemic, and in the post-pandemic world, it is crucial to reflect on the lessons learned. Achieving the broadest possible vaccination coverage has been perceived as an effective way to end pandemics. Yet, vaccination hesitancy—reluctance or refusal to vaccinate despite vaccine availability—has been identified as one of the top threats to global health.^1^ Vaccination coverage rates for COVID-19 vary in different parts of the world and regions of each country.^2^ Previous research has also spotlighted a lower vaccination uptake among ethnic minorities within a country, as well as persons with mother tongues other than the majority.^3^ These factors are pertinent to migrants. Broad vaccination coverage, including in vulnerable populations, is crucial in protecting public health and combating health inequalities.

Since 2022, a new crisis—the war in Ukraine—has shadowed Europe. The war has led to millions of persons fleeing war and seeking international protection in Europe amid the pandemic. Before the full-scale invasion, only approximately 40 per cent of the adult population in Ukraine was vaccinated against COVID-19.^4^

Public health needs for broader vaccination coverage and the possibility of future co-existent crises raise essential questions about authorities’ obligations regarding protecting public health through vaccination. They also beg for reflections on crisis preparedness. This article will analyse the legal obligations related to protecting the right to health in addressing vaccination barriers among newly arrived adult migrants. The article will thus go beyond the traditional ‘to force or not to force’ approach taken in legal scholarship, which often discusses whether compulsory vaccination is a permissible infringement on the right to privacy,^5^ and instead will analyse broader obligations related to the realization of the right to health.

The article’s aim will be addressed through the following tasks. First, Section II will analyse and map out the material substance of international human rights obligations concerning vaccination as part of the right to health, as delineated by the United Nations and Council of Europe. As a result of the analysis, the framework for assessing compliance with human rights obligations concerning vaccination will be clarified. The obligations will be summarized in Table 1. Secondly, the article will test the framework of one national legal system, Sweden, a country with generally high vaccination coverage and trust in vaccines. Section III examines how human rights obligations were transposed into national law and where the gaps still exist. Due to the co-existence of crises, the section will also analyse how the situation with COVID-19 vaccination of newly arrived Ukrainian refugees has been addressed. Furthermore, the interview study conducted with Ukrainians displaced in Sweden due to the invasion will be presented. The interview study will allow reflection on the hindrances to COVID-19 vaccination perceived by Ukrainian refugees in Sweden and how recipients perceive the fulfilment of the obligations by public authorities. Addressing these tasks requires a combination of different research methods. Sections II and III will explain the sources used to answer the research questions. Section IV concludes.

## II. VACCINATION WITHIN INTERNATIONAL HUMAN RIGHTS FRAMEWORKS

This section will analyse the material substance of international human rights obligations concerning vaccination as a part of the right to health. Several of the Council of Europe and United Nations human rights treaties have laid down the right to health and these include:

- Article 11 European Social Charter (revised) (ESCr);
- Article 12 UN Covenant on Economic, Social, and Cultural Rights (ICESCR);
- Article 5 (e)(iv) UN Convention on the Elimination of All Forms of Racial Discrimination;
- Articles 11 and 12 UN Convention on the Elimination of All Forms of Discrimination against Women;
- Article 24 UN Convention on the Rights of the Child (CRC); and
- Article 25 UN Convention on the Rights of Persons with Disabilities.

All the conventions mentioned above have treaty bodies that may provide decisions in a specific case and comments on state’s reports; the committees can also issue statements concerning how the provisions of the treaties should be interpreted (so-called general comments, recommendations, or statements of interpretation). In this section, the decisions, comments on the state’s reports, and interpretative views of the treaty bodies to the above-mentioned conventions will be analysed to establish how they construe the substance of the obligations related to vaccination when such duties cannot be specified through the general rules of interpretation, which is mostly the case with issues related to vaccination. The UN Official Document System and HUDOC databases were used to search with the search terms ‘immunization’, ‘immunisation’, and ‘vaccine’, which yielded over 500 documents.

Prevention and control of epidemics and endemics are obligations directly established in international human rights treaties, particularly in Article 12.2(c) ICESCR and Article 11(3) ESCr. The advancement of modern science allows counting vaccines to be a powerful tool for preventing epidemics. Access to vaccination has long been considered an integral part of the right to health, and treaty bodies regularly request states to inform them about vaccination rates and encourage higher rates (particularly among migrants).^6^ The possibility to receive or refuse vaccination is not directly recognized as a human right but is part of the complex human rights framework.^7^

The right to health, as an entitlement for the population, is progressively realizable, meaning that states have obligations to take steps to, for instance, prevent epidemics by acting to the maximum extent of their available resources by all appropriate means. Every progressively realizable right also includes so-called ‘core’ obligations, non-derogable obligations of strict legal liability.^8^ The Committee on Economic, Social and Cultural Rights (CESCR) considers that the duties to establish immunization programmes and to take measures to prevent, treat, and control epidemic or endemic diseases are obligations of priority, though these obligations are not listed as ‘core’ ones.^9^ Due to the prioritization of this area, low vaccination rates—often referred to as those below 95 per cent, or such as recommended by the World Health Organization (WHO)—have been criticized in the practice of various treaty organs.^10^ The vaccination coverage should aim not only to reduce the disease frequency but also, if possible, to neutralize the reservoir of infection.^11^ This emphasizes that vaccination is necessary from the public health perspective and should not be seen only as an individual right.

In 2000, the CESCR produced General Comment No. 14 on the right to health. This comment became an authoritative source for interpreting the right to health obligations. The General comment clarifies that the right to health has four crucial elements—availability, accessibility, acceptability, and quality. The framework is often referred to as AAAQ. Although treaty bodies for other international conventions do not necessarily refer to the framework, its elements are implicitly present in practice on vaccination—as will be illustrated further in this section.^12^ The obligations that arise through the elements concerning vaccination are explained below.

The element of *availability* relates to determinants of health (including medicine, healthcare professionals, and services) being sufficiently available to satisfy the population’s needs.^13^ The availability of vaccines has been highlighted in the practice of treaty bodies. For instance, the treaty bodies indicated the necessity to create widely available vaccination programmes for various groups, including migrants.^14^ The Committees have urged raising expenditures for vaccination programmes to increase the number of health centres, professionals, and vaccines available.^15^ Research on vaccines should be promoted and funded to enable their availability (and quality).^16^ The obligation to make different ranges of vaccines available was also stressed.^17^ To enable the availability of vaccines, the treaty organs encourage mobile arrangements and community-based efforts.^18^ However, the existing case practice on the element of availability in vaccination has not significantly focused on migrants, except for the availability of vaccination programmes.

*Accessibility* implies that vaccinations are available without discrimination to all, especially to vulnerable populations, within physical reach and are economically affordable and informationally accessible.^19^ Concerning physical accessibility, it has been stressed that immunization programmes should be accessible in different health districts, including rural and mountainous areas and conflict zones.^20^ Economic affordability was explicitly highlighted for vulnerable groups.^21^ Affordable access to vaccines in a community is considered a ‘core’ obligation.^22^ Providing regular, factual, and reliable information about immunization to the public has been deemed necessary to make vaccinations informationally accessible, particularly in languages migrants understand.^23^ The committees also demand that the gaps in immunization be identified to increase accessibility.^24^

Regarding non-discrimination, the treaty organs recommend that states ensure equal access to vaccination for ethnic minorities, migrants, persons with disabilities in different regions of a country, and disregarding social status.^25^ This is to be achieved, in particular, by making immunization programmes accessible to the broad population.^26^ Healthcare professionals should also be trained to aid in ending discrimination and segregation of minorities.^27^ An example of the reasoning for discrimination in the question of access to vaccination can be found in the European Committee of Social Rights’ (ECSR) case of *Médecins du Monde—International v France*. The ESCR found a violation of the freedom from discrimination, mainly because the government has not made any targeted measures to address the high levels of preventable infectious diseases among Roma.^28^ The Committee observed that no health education was provided, and no actions were taken to address the known problem of distrust in the healthcare system.^29^ Treating migrants with known issues related to access to immunization in the same manner as the rest of the population, when their position is different, constitutes discrimination.^30^

*Acceptability* means that vaccination respects medical ethics and is culturally appropriate for individuals and groups, particularly to improve health.^31^ Acceptability appears to be connected to a certain extent with informational accessibility: The treaty organs require states to provide extensive information about vaccination to make it culturally acceptable. The treaty organs are, in particular, concerned with attitudes towards vaccination in a specific society.^32^ Therefore, they supervise whether the immunization awareness-raising campaigns are developed,^33^ whether states support public advocacy, and whether vaccination is encouraged through the media.^34^ Another concern for treaty bodies is whether the measures to establish or restore public trust were taken^35^ or whether educational health programmes about the immunization function were created or improved.^36^ Attention has been paid to disparities in vaccination rates within a country. The treaty bodies noted that immunization awareness-raising campaigns should specifically target certain ethnicities or geographic locations when gaps in vaccination coverage are identified.^37^ This requirement interconnects the element of acceptability with the non-discrimination element discussed above.

The element of *quality* requires that the states provide scientifically approved medicines of good quality and ensure that skilled medical professionals provide medical services.^38^ Not many aspects of the element of quality were lifted in the case practice. Among those that were, the treaty organs were concerned that responsibility for vaccine storage and keeping track of vaccination dates was delegated to private persons, such as parents. The treaty organs considered that these factors could impact the quality and put poor households in a particularly disadvantageous position.^39^ The Committee on the Rights of the Child also emphasized the importance of properly functioning the cold chain to preserve good quality vaccines.^40^ Otherwise, general recommendations were provided to improve the quality of preventive interventions, including vaccinations, unrelated to being a migrant.^41^

It is possible to conclude that immunization of the population, including migrants, has been established in case practice as an intrinsic part of the right to health. Through the AAAQ elements of the right to health, the duties of the states can be specified. These are summarized, grouped, and numbered in Table 1. The numbered obligations in Table 1 will be referred to further in the article.

**Table 1:** . Positive obligations as to vaccination

Availability	Accessibility	Acceptability	Quality
1. Increase expenditure for vaccination programmes to increase the number of health centres, professionals, and vaccines available	**5. Make vaccinations economically affordable** (+)*	14. Promote encouragement, particularly through media, and support public advocacy on vaccination*	15. Ensure that the population is immunized with scientifically approved medicines of good quality
2. Fund and promote research on vaccines	**6. Provide and improve health education about immunization**		See 2 in the Table 1.
**3. Ensure that widely available and accessible vaccination programmes for various groups are established** (+)			16. Ensure that skilled medical professionals provide medical services
4. Make different ranges of vaccines available*	**7. Identify the gaps in immunization** (±)		17. Assume responsibility for storage and tracking vaccination dates (±)*
	**8. Address the problems and gaps with immunization through tailored and targeted measures** (±)*		18. Ensure the proper functioning of the cold chain to preserve good quality vaccines
	9. Address distrust, establish, or restore public trust in the healthcare system or other known vaccination-related problems*		
	**10. Develop awareness-raising campaigns that target certain ethnicities or geographic locations when the gaps in vaccination coverage are identified**		
	**11. Provide regular, extensive, factual, and reliable information about immunization, particularly in languages migrants understand** (±)∗		
	12. Ensure vaccination is geographically accessible and encourage mobile arrangements and community-based efforts*		
	**13. Train healthcare professionals to aid in ending discrimination and segregation of minorities**		

Most of the duties identified in the practice of human rights treaty bodies are relevant to migrants, including newly arrived ones. The practice concerning the immunization of migrants predominantly concentrates on accessibility and acceptability elements, described as obligations 3, 5–14 in Table 1 and marked bold. The elements of accessibility and acceptability are also often interconnected in the practice of vaccination (see obligations 3, 7–11 in Table 1, see also 2 concerning availability and quality).

The state obligations concerning vaccination are not formulated as particularly strong. The duties related to vaccination are not considered a minimum core, except for the obligations of economic affordability. The fulfilment of the state’s responsibilities can often depend on the available resources, emphasizing the importance of accountability for the resources spent.

## III. APPLYING FRAMEWORK: THE SWEDISH CASE

### A. Legal responsibilities for vaccinating migrants in Sweden

Section II identified the obligations related to the immunization of migrants as part of the right to health. These are primarily associated with accessibility and acceptability and were summarized as obligations 3, 5–14 in Table 1. Now, I will test the framework of Sweden’s legal system to determine to what extent obligations 3, 5–14 were transposed into national law. To visualize the findings, when similar obligations are found in the system, they will be marked with (+) sign in the table. If obligations do not entirely coincide, they will be marked as (±).

The identification and analysis of legal obligations in Sweden will be conducted per the hierarchy of legal sources accepted in Swedish law. This means studying legislation, preparatory works, case law, and legal doctrine. To exemplify the challenges with interpreting the legal obligations, I also requested public information from the authorities regarding measures taken to vaccinate migrants.

The Swedish legal system is dualistic, which means that international treaties are not directly applicable unless implemented as domestic legislative acts.^42^ International treaties can be relevant to the interpretation of domestic law when authorities, including courts, decide that gaps in national legislation can be filled in through treaty-conforming interpretation.^43^ With the exceptions of the CRC and the Convention for the Protection of Human Rights and Fundamental Freedoms (ECHR), most of the treaties discussed in the previous section do not have the status of domestic law.^44^

The international human right to health has been reflected in the national legal system in several ways. Chapter 1, Article 2 of the Instrument of the Government, one of the Sweden’s constitutions, establishes that public institutions shall secure favourable conditions for good health. However, the provision was designed not to lay down a justiciable right but a broad purpose that authorities should strive for.^45^ The right to health reiterations are also visible in healthcare legislation. Here, the legislator stated that the purpose of healthcare is good health and care for all the population. This purpose can be seen as an aspiration to progressively realize the right to the enjoyment of the highest attainable standard of health in future. The measures to be completed and the supervision of whether the goals have been measurably achieved are not specified. I will now delve into the question of the division of responsibilities for vaccination as part of the right to health in Sweden.

The Swedish administrative model is characterized by the high independence of authorities from the Government: Authorities are separated from the Government, and the Government or other authorities cannot decide on behalf of the authorities how to interpret the law or exercise the powers in each case.^46^ The Government is a collective decision-making body, and the ministries’ powers to decide are limited. The Government provides general directions, which the authorities should follow, but these directions should not concern individual cases.^47^

Regarding vaccination, the Government, for instance, has provided additional funding to make vaccines against COVID-19 available for newly arrived persons from Ukraine. It thus attempted to address obligations 3 and 5, as numbered in Table 1, regarding making the vaccination programmes available and economically accessible to migrants.^48^

The responsibility for vaccination and welfare of migrants with temporary statuses resides upon various authorities, which will be described below.

The Public Health Agency of Sweden is a central authority that coordinates and implements infectious disease control measures.^49^ The Agency has broad obligations concerning achieving equal health for the population and is supposed to provide various kinds of expert support to the Government.^50^ The Public Health Agency’s assignments concerning vaccination are further specified as evaluating the effects of vaccinations. This duty appears to be connected with, though not identical to, obligation 7 in Table 1 to identify the gaps in vaccination coverage (the assignment to identify the gaps is not specified but can be a part of the vaccination effect evaluation).^51^ The Agency is also responsible for the national vaccination registry. From 2021, the registry includes information about COVID-19 vaccination, though not necessarily about persons with temporary migration statuses.^52^ To fulfil the assignments laid upon the Agency, it produced a National plan for vaccination against COVID-19, which mentioned the need to reach out to groups with lower vaccination rates (also related to obligation 7 in Table 1).^53^ In March 2022, it issued recommendations concerning the infection disease control measures for persons arriving from Ukraine, which underlines low vaccination coverage in Ukraine.^54^ Some general information about vaccinations was translated into Ukrainian and Russian, though one has to actively search for the information on the website. The Agency’s translations engage obligation 11 in Table 1 on providing information on vaccination in accessible languages, though the regularity and extensivity of the information for migrants can be questioned.^55^ The Public Health Agency has produced many recommendations on various vaccination issues, such as limiting the possibility of patients choosing a specific vaccine among the approved ones.^56^ The latter recommendation can be seen as retrogressive to obligation 4 in Table 1 on enabling access to different ranges of vaccines.^57^

The Migration Agency is also responsible for the welfare of persons granted temporary protection and asylum-seekers. Such responsibilities explicitly include issues of economic support for migrants in temporary situations and providing them with places to live.^58^ Those migrants with temporary status who do not have means can receive economic support of up to 71 Swedish kronor per day for adults living on their own and up to 61 kronor for those adults living with their family to fulfil their daily needs, such as food, transport, clothing, and so on.^59^ Generally, the Migration Agency should provide information to asylum-seekers about their rights and obligations (which can relate to duty 11 in Table 1). The legislation does not specify such an obligation concerning persons granted temporary protection.^60^ However, the Migration Agency has factually informed about the possibility of receiving vaccination. The information was provided in the form of print-out materials when applications for temporary protection were submitted in person and on the webpage when the application was submitted online.^61^ The web version of the information offers a paragraph of text with recommendations to obtain COVID-19 vaccination free of charge for the group. In contrast, the written version of the text did not contain such information.^62^ It is available on the website for anyone to read; however, it is necessary to search for such information actively.^63^ Thus, the Migration Agency has taken upon certain responsibilities related to obligation 11 by providing factual information about immunization in languages migrants understand. The obligations exercised do not extend to the information being regular and extensive.

The primary responsibility for delivering healthcare services to residents, including vaccination, lies with the 21 county councils in Sweden.^64^ Migrants with temporary status, such as those granted temporary protection, are not considered residents in the meaning of Swedish healthcare legislation and, until the summer of 2024, could not receive Swedish personal numbers.^65^ For these migrants with temporary status, special legislation imposes obligations on country councils to provide some healthcare services. These should at least include so-called care that cannot wait, one medical screening, and certain reproductive care, but the county councils can decide to broaden their own responsibilities.^66^ Care that cannot wait is usually defined as care above the emergency one, necessary to prevent death, serious deterioration of health, or treatment becoming significantly more prolonged and expensive.^67^ Vaccinations, as a general rule, do not fall within the definition of care that cannot wait. However, upon the suggestion of the Swedish Association of Local Authorities and Regions, the county councils provided the vaccination against COVID-19 free of charge for migrants with temporary status.^68^ As specified earlier, in conjunction with governmental funding, making vaccination against COVID-19 free of charge for the whole population engages duties 3 and 5.

Migrants with temporary status lack personal numbers, which makes documenting vaccinations difficult. For some time, such migrants could not obtain the certificates; however, in 2022, receiving the vaccination certificates on paper became possible. This change relates to, though not entirely coinciding with, obligation 17 in Table 1 regarding keeping track of vaccination dates.

Vaccinations in Sweden are classified as healthcare services and infection disease measures, which result in a complex regulation of the matter.^69^ Infection disease control legislation requires each county council to appoint a special actor—infection disease officers.^70^ These actors can act as independent authorities in matters concerning the exercise of public powers or as a part of the county councils in other matters, such as providing information or advice.^71^ The infection disease control legislation recognizes that county councils, through infection disease officers, must inform the public and advise on how the population should protect itself from infectious diseases, including through vaccination (this can be seen as a part of the obligation 11 in Table 1).^72^ However, explicit obligations to provide health education, identify and address vaccination gaps through tailored and targeted measures, address distrust, conduct targeted awareness-raising campaigns for vulnerable groups, translate information to the languages migrants understand, and ensure geographic accessibility of vaccinations are not foreseen in domestic law (see, in particular, obligations 6–14 in Table 1).

The legislator provides broad leeway to the county councils on how to realize the right to health by vaccinating migrants with temporary status. As mentioned above, to find out how the obligations have been implemented, I have sent letters to all 21 county councils, requesting public information on the measures taken for vaccination against COVID-19 for people who have received a residence permit due to the full-scale invasion of Ukraine. In addition, I requested that similar information be provided about asylum-seekers in general and measures to ensure access to information and advice for those who cannot speak Swedish.^73^ Seventeen of the county councils offered some answers to the questions posed. However, some pointed out that they cannot describe all the measures taken during such a prolonged period in a letter and/or that measures were not necessarily documented as public information.

From the answers obtained, it is possible to observe that the flexibility of the legislation resulted in different realizations of the obligation to inform the migrants in Swedish county councils. Several county councils have only written general information on vaccination for migrants on their websites and also in Swedish.^74^ Some responded with booklets and posters in several languages.^75^ Some county councils have been actively investing in outreach activities. In several cases, such activities were primarily placed at the special units, providing healthcare for displaced persons.^76^ Sometimes, the information was provided when invited to the medical screenings for public health grounds and in language schools for foreigners.^77^ Known civil organizations were informed about the vaccination, including through translators and cultural translators. Some have employed healthcare communicators, health guides, and cultural interpreters for awareness-raising activities or co-working with communicators from other county councils.^78^

Occasionally, vaccinations were booked at the places where newly arrived migrants lived.^79^ Since migrants with temporary status were not Swedish residents and lacked Swedish personal numbers, they could not book vaccination appointments via electronic systems. For this reason, drop-in and telephone lines were available for booking in some county councils.^80^ In one county council, information about the booked times for vaccination has been sent to the addresses where temporary migrants reside; however, due to the migrants being moved to other locations, the letters did not reach them, and the measure was not deemed as particularly successful.^81^ The county council then tried to heighten the vaccination rates through outreach activities in the towns where most refugees lived. The county council assessed the geographical proximity of the vaccination centres next to the Migration Board to be a more successful measure.^82^

Thus, the approaches taken by the county councils within the country are dramatically different: from not focusing on the specific group other than providing general information on the websites to active attempts to reach the group via various means.

Providing overall reflections on the legal system, it is possible to conclude as follows. Although the right to health is reflected in Sweden’s legal system, it is formulated broadly. The general purpose of good health, read together with similarly broad and patchy obligations of authorities, does not allow specification as to who has the responsibilities for every component of the right to health regarding the vaccination of migrants, as identified in Section II (see Table 1). Some obligations, such as those related to the economic affordability and availability of vaccines for the whole population, have been created *ad hoc* (duties 3 and 5) during the COVID-19 crisis. Others, such as identifying the gaps in vaccination coverage (obligation 7) and providing information about immunization (obligation 11), have only been partially reflected in Swedish law. Obligations 3, 5–14 concerning the vaccination of migrants and the accountability for their fulfilment are not fully specified in domestic law.

The county councils’ obligations in the national legal system are addressed broadly: as public authorities in general, they shall strive to secure the conditions for good health, especially those related to vaccination as a healthcare service. The study shows that they address their duties differently, which results in the right to health being dependent on where migrants reside in Sweden. From communication with authorities, it is impossible to estimate whether the treaty-conforming interpretation principle played any substantive role in their reasoning on understanding their obligations regarding the immunization of migrants.

On the one hand, the absence of overly specific requirements regarding how the positive obligations concerning vaccination as part of the right to health should be fulfilled allows authorities to implement flexible measures that can be adjusted to the specific situation, which is visible when discussing how different county councils approach the issue in practice. On the other hand, the study clearly shows that not all elements of the obligations—as discussed in Section II (see Table 1)—are being comprehended, reflected, or fulfilled in the legal system. The absence of specified functions for authorities—such as those delineated in Table 1—may hinder preparedness for future health crises and make vulnerable population groups even more vulnerable. According to Bennett and Carrey, the transparency of the legal framework is key to an effective response to public emergencies; the absence of clarity can obstruct the effectiveness of the response.^83^ Here, the absence of specified functions concerning responsibilities for vulnerable groups and the lack of a mechanism for accountability assessment can lead to problems with effective responses. This concern is relevant not only for migrants but for various population groups.

### B. Barriers to vaccination among Ukrainian refugees: interview study

Section III.A showed that in Sweden, the legal obligations concerning the vaccination of migrants are often blurred and regulated in a complex manner. Only some obligations, marked in Table 1 as (+) or (±), have been engaged in Swedish regulations during the COVID-19 crisis. In this section, with the help of the interview study, I will reflect on the barriers to vaccinations experienced by Ukrainian refugees living in Sweden. This may provide indications regarding the way the obligations reach their recipients. In case the interview study indicates dissatisfaction with reaching recipients’ specific obligations, these are marked as asterisks in Table 1. The reflections, however, have limited general value and are primarily given as a methodological example of conducting research on vaccination as part of the right to health. This interview study focuses on barriers to obtaining COVID-19 vaccinations, and the hindrances to obtaining other vaccines may be different. The study has a limited sample size and may be non-representative. Its focus on Ukrainian refugees in Sweden does not characterize all refugees in general.

The interview study was conducted in Scania County Council, one of the county councils that actively worked to inform the migrants about COVID-19 vaccinations by engaging health communicators, providing information in different languages at health centres specialized in migrants, and working with so-called information hubs.^84^ The study included persons who had obtained temporary protection in Sweden, all of them were citizens of Ukraine. For participants’ recruitment, the advertisement about the study was distributed at the premises of the Ukrainian centre in Lund, on the Facebook pages of Ukrainians in Scania (Malmö and Lund), and on Telegram channels. Some participants were recruited by snowballing.

The interviews were semi-structured. They included 34 open-ended questions covering topics such as socio-economic living conditions in the home and host country, risk appraisal, anticipated regret, vaccine confidence, motivation, past behaviours, social networks, accessibility, sources for obtaining information, including social media, thoughts, and feelings related to vaccination in general and COVID-19 pandemic and vaccinations. The study by Brewer and others on the psychological processes to improve vaccination coverage was used as an inspiration to construct the questions.^85^ Other researchers were engaged in creating the questions.^86^ The questions were partly used in other survey studies on vaccination willingness in Sweden.

To make interviewees comfortable and working conditions safe, the participants were suggested to meet at my office, in public places where sensitive conversation was possible, or online. The interviewees usually opted for face-to-face interviews at the interviewer’s office or small libraries, except for one digital interview in Zoom. Upon meeting, I provided general information about the research project and the purpose of the interview. The consent form in Swedish, Ukrainian, or Russian was presented, and each point was discussed. I asked participants if they had any questions before signing the form and explained that should the question arise, they were welcome to ask at any point in time. All participants expressed concerns that their opinions might not be interesting for the study and that they might have forgotten some information. I assured them that whatever their opinion was on vaccination, it was important to the study and that it was normal not to remember all events. I also informed participants that they could decide to pass some questions if they felt uncomfortable with them, interrupt the interview, and leave at any time. I asked if participants wanted to have an interview with the doors closed and ensured that the person knew how to leave the facility. I suggested that if participants remembered some answers or events later during the interview, they could return to previous questions. The interviews lasted between 40 and 180 min.

During the interviews, some participants showed signs of psychological distress, especially when the questions as to when the persons came to Sweden were asked. In these cases, the participants were asked whether they needed to pause or to interrupt the interview; water was also suggested. In all the cases, the participants resumed the interviews.

In total, 15 people who used to live in different parts of Ukraine were interviewed. The participants’ ages varied from 26 to 76. Fourteen women and only one man were interviewed (see Table 2). They had been staying in Sweden for between 4 and 11 months at the time of the interview. Some interviewees had started working in Sweden, whereas others had not. Most of the participants attended Swedish language courses. The interviewees lived in different parts of Scania County Councils.

**Table 2.** Characteristics of the study participants

Variables	Participants, *N* = 15
Age, years	26–76 Average 47
Women	87% (*N* = 14)
At least 4 years of higher education at a university	100% (*N* = 15)
Vaccinated before the full-scale invasion with two doses (vaccinations between July 2021 and December 2021)	87% (*N* = 14)
Vaccinated in Sweden after the beginning of the full-scale invasion	27% (*N* = 4)

All the interviews were conducted in Ukrainian or Russian, depending on the interviewee’s choice. Saturation was achieved when no markedly new ideas or opinions were expressed during the last four interviews. I translated and transcribed all the interviews into English. The analysis of the interviews included the following steps. First, the transcriptions were read to become more familiar with the materials. Secondly, the overarching themes were identified, considering the previous discussion on the state’s obligations. Thirdly, the materials were indexed manually, and every theme was identified. Fourthly, the data were placed in the framework of the current article and described. Therefore, the method can be characterized as the thematic analysis of the interviews.

Most interviewees had obtained two doses of the vaccine in Ukraine (see Table 2), which appears to be an overrepresented group, considering the generally low vaccination rates before the war. At the time, the Public Health Agency recommended receiving at least three doses of vaccine, and similar recommendations were valid in Ukraine. However, only four participants had obtained an additional recommended dose of vaccine in Sweden, despite vaccination being offered free of charge at the time.

The interviews provided the following overarching themes on vaccination barriers:

1. lack of informational accessibility related to obligation 11;
2. lack of accessibility due to the booking system constraints related to duty 8;
3. lack of economic accessibility related to obligations 5 and 12;
4. lack of acceptability due to other priorities, which relates to obligation 14;
5. lack of acceptability due to the absence of information about own health and care in case of side-effects, engaging obligations 8, 9, and 11; and
6. lack of acceptability due to the documentation process, engaging duties 8, 9, and 17.

The issues raised within these themes during the interviews are described below.

As to the first theme—the lack of informational accessibility—most participants have reported that they had never obtained any information about vaccination from national authorities in Sweden or did not remember ever obtaining such information. Those who said they did not recall receiving information clarified that they had gotten many documents in various languages, the content of which was difficult to remember. Information overload, difficulties remembering information, and forgetfulness due to stress were issues raised in all the interviews. Accessibility of information due to language was highlighted: many migrants spoke Ukrainian and Russian, and information in other languages was inaccessible. They underlined attempts to translate information through various translation software (primarily Google Translate) and difficulties interpreting the translation results. Most interviewees did not know where or how to obtain a vaccination in Sweden—whether all healthcare providers or only vaccination centres provided, and whether one needs to travel to the municipal centre to receive a dose. A few did not know whether the vaccines were provided free of charge. They assumed that vaccines were not considered ‘care that cannot wait’.^87^ The migrants who did not know about vaccination against COVID-19 expected the information to be provided when they were called for medical screenings, which they had not yet been called to at the interview. The knowledge of vaccinations was primarily based on what they had heard from other migrants, read in online social networks for Ukrainians in Sweden, and sometimes from personal encounters. Only one person could understand the information about vaccination provided on public transport. The Swedish news channels were not followed. The results of the interview study thus allow reflection that, although the obligation to inform was to some degree reflected in national law and in the practice of the county council (duty 11 in Table 1), the interviewees did not view that they received information regularly or extensively in a language understandable for them.

The second theme was the constraints related to booking time for vaccination. The interviewees’ situations have been different: some lived alone, some had children to care for, and some worked or studied full-time. The interviewees highlighted that they had been used to a convenient booking system for vaccination in Ukraine, where they could choose vaccines and locations. However, due to the absence of a personal number, booking online—the option most were used to—was not available to them in Sweden. English-speaking migrants explained that telephone booking queues were incompatible with their jobs or other obligations. Non-English-speaking migrants often could not book time via telephone due to language constraints. They were concerned that they needed to ask someone else to book the time for them, such as relatives or friends in Sweden, or disclose sensitive information. These have been limiting their choice. Persons were reluctant to search for drop-in options because of the need to work or study and the unforeseeable waiting time for them. Two of those vaccinated in Sweden had relatives in the country, and one was English-speaking. All those who received vaccination in the country were unemployed at the time of vaccination. Here, it is possible to reflect that the duty to provide tailored measures for the group has not reached the recipients (obligation 8). This tailored means could have been realized in particular by making telephone choices available in other languages or allowing for electronic booking without using personal numbers.

Thirdly, the interviews yielded that despite vaccination against COVID-19 being offered for free, it was not always economically accessible for participants. The migrants who were unemployed and lived on the economic support provided by the Migration Agency in smaller towns and villages considered it to be too expensive to travel to obtain the vaccination. Public transportation was free for several months after activating the EU Temporary Protection Directive in the county council. But, during the interviews, the participants had to pay for public transportation. Participants described that buying a round trip ticket to the municipal centre, where vaccination was available, cost more than the daily allowance they received from the Migration Agency (the price of the ticket was estimated to be over 80 kronor, compared to a daily allowance up to 71 or 61 kronor per adult). The prophylactic measure of vaccination was not as important as access to food, and the price of the tickets constituted an explicit barrier. This discussion engages obligation 5 in Table 1 but sheds new light on it. Despite immunization being offered free of charge, it was still considered economically inaccessible for some migrants due to the socio-economic conditions in which they lived. It also shows that the duty can be interrelated with the obligation to make immunizations geographically accessible (duty 12) and can be interrelated with the physical distance and the cost to reach it.

The fourth theme highlighted in the discussion was that many regarded vaccination as an unacceptable service due to not being encouraged enough to have it. Though all participants, except one, acknowledged that vaccination could be essential and they would most likely vaccinate in future, it was underlined that those who have not been vaccinated in Sweden did not prioritize the benefits for their own health at the moment of the interview. An elderly participant who was previously afraid of COVID-19 and vaccinated as soon as vaccines became available but reluctant to vaccinate with the third dose explained it as follows:

The attitude towards life is contextual; it changes depending on where we are in life. Some things are more horrible than COVID. When small children die, when the buildings are blown by shelling… No, I do not think about my prospects of dying of COVID now. It does not matter to me now. I am not sure that it is important for someone.

Many participants repeated this line of reasoning: some emphasized that sense of guilt for having a normal life, compared to those who were left behind: it was taboo for them to think about improving their health while those who stayed in their home country struggled with survival or died. Participants wanted to know that vaccination was vital or meaningful for society to be acceptable to them. Other participants considered building a new life a priority and said that they could not spend time on vaccination. In particular, when discussing the possibility of coming for a drop-in for vaccination or booking time online, it was emphasized that such options are not necessarily feasible since drop-in waiting times were perceived to take several hours. The common side effects of vaccination, such as fever and general weakness, were also perceived to be not acceptable in the situations persons live in since it could reduce their income, restrict the educational process, result in not having a carer for children, or—it was not acceptable to feel sick and down in the premises where several other people live. Prioritizing earning money or studying was stressed in several interviews. Here, it is possible to reflect that the migrants did not feel encouraged enough to prioritize vaccination against COVID-19, among other concerns. The theme indicates that obligation 14 did not necessarily reach this group.

The fifth theme relates to the lack of acceptance due to the absence of information about one’s health and side effects. Here, the participants perceived that they could not accept vaccination because they did not have enough information about the need for vaccines. Some expressed concern about their weakened state of health due to previous colds and being unsure whether they had COVID-19 recently. They wanted to get access to the information about their level of antibodies to decide, but this kind of medical screening was not available, and therefore, the vaccination was not believed to be necessary. Interviewees wanted vaccination decisions to be individually tailored to their disorders and state of health. Another issue was the absence of the possibility of choosing which vaccines would be injected—as specified earlier in Section III.A, the Public Health Agency limited the possibility of selecting the vaccines (a decision that the Parliamentary Ombudsman later criticized). Most of the participants expressed concerns that some vaccines were better than others. In Ukraine, they could select vaccines and felt responsible for the choice, and it was unacceptable to have some other vaccines (indicating dissatisfaction with obligation 4). Several participants expressed concern about the side effects. As to more serious side effects, the participants were worried about receiving care in Sweden because they were entitled only to certain limited healthcare (‘care that cannot wait’). Here, it is possible to observe that several obligations did not quite reach the recipients: these are related to the distrust or establishing trust in the healthcare system because participants did not feel they would receive necessary care in case of side effects (duty 9 in Table 1), and, to some degree, access to information about one’s own health (obligation 11). The measures were also not perceived to be tailored to the individual needs of participants (obligation 8).

Some participants highlighted that they could not access the electronic version of the vaccination certificates due to the absence of a personal number. When discussing why the electronic version of the certificate is essential and not the paper version, one participant said:

I am considering going to Ukraine to get vaccinated because I can get my certificate in ‘DIYA’ [electronic state services in Ukraine] and have it everywhere with me. I have learned so far in the war that paper is unreliable.

Another woman explained that when she requested the paper version, she did not receive it. Strict limitations in Ukraine during 2020–2021 due to the pandemic defined the need for documentation to visit various facilities showing the vaccination passport (pharmacies, public services or shops). Similarly, it was not possible to travel abroad. Although similar limitations were not established in Sweden, the participants highlighted that having the documents ‘just in case’ is crucial. These reflections of the participants are connected with obligation 17 on the responsibility of keeping track of vaccination dates, but also indicate some need for more tailored measures (duty 8) and distrust of different healthcare systems in validating documents from each other (relates to duty 9).

The participants also came up with various suggestions to increase the availability and acceptability of vaccinations for them. Some expressed the need to communicate with them via Facebook and Telegram, and to send letters with invitations for vaccination or information about it. It was suggested to provide vaccinations where Ukrainians live, vaccinations at the dormitories or Ukrainian centres, or to send invitations with tickets for public transport. Making information more accessible as to the benefits of vaccination and its effects on the body, with scientifically proven facts, through video or written communication, was recommended by many interviewees.^88^ The opening up of possibilities for electronic communication with people who do not have personal numbers was stressed, as well as having drop-in options, particularly during non-working hours. The possibility of receiving electronic vaccination passports was also assessed as necessary.

To sum up, the discussion in this section illustrates how migrants experience vaccination barriers. The interview study points out that Ukrainians in Southern Sweden primarily experience barriers to the accessibility and acceptability of COVID-19 vaccinations. The interview study demonstrates that participants were hesitant to vaccination in particular because of the obligations related to economic affordability pertaining to public transportation (duty 5) or geographic accessibility without it (duty 12), informational accessibility as to the procedure in the host country, the regularity, and extensivity of the information in the languages the migrants understood (duty 11). Here, the measures taken (making vaccination free of charge and translating some information) did not suffice to enable the group to undertake immunization. The information about vaccination against COVID-19 has either not reached the participants or has been forgotten by them. The interview study also demonstrates that efforts related to encouragement of vaccination in the life situations of migrants (14), tailoring measures for their language capabilities and the absence of personal numbers when it comes to booking and obtaining the certificates, and addressing the distrust (8, 9, and 17) were not perceived as substantial in enabling immunization.

The interview study indicates that legal constructs are often seen as a barrier to vaccination. These include the limitation of care to ‘care that cannot wait’, limitations of the possibilities to choose vaccines, non-recognition of residence status, and, with it, the absence of personal numbers and the possibility of obtaining an electronic certificate.

## IV. AT THE READY TO… FALL BETWEEN THE CRACKS?

Immunization, including for migrants, has been established as part of the international human right to health. The obligations related to vaccination shall be implemented gradually, depending on available resources, and are rarely considered part of the ‘core’ obligations, except for economic affordability. The study allowed the specification and mapping out of the obligations expressed in the case practice of the international human rights treaty bodies in Table 1. Table 1 can serve as a framework for critically analysing national laws regarding where the gaps in fulfilling these obligations exist. As seen, the obligations on vaccination relate to all elements of the right to health, but in relation to migrants, the availability and acceptability aspects were marked specifically in the studied materials. The obligations related to the vaccination of migrants were marked in bold in Table 1.

The framework was tested within Sweden’s legal system. The study showed that the obligations on immunization, including vaccination of temporary migrants, become dispersed and non-specific nationally. Although the right to health is laid down in the Swedish legal system, its realization concerning vaccination is not specified in the legal acts and is thus broad. Many authorities are authorized to work with various aspects of vaccination in general, as well as different responsibilities for migrants’ welfare. The system’s design does not specify responsibility for each component of the right to health regarding the vaccination of migrants, which becomes everyone’s and no one’s issue. The extent of responsibilities for implementing each of the components of the right to health to ensure the vaccination of the vulnerable population is not transparent, which can lead to the ineffectiveness of the legal system in a crisis. The problem with the transparency of the function is visible by indicating that different county councils handled the situation differently, from providing the minimum amount of information to actively attempting to find and fill in the vaccination gaps through various means. Using Table 1, it was possible to see that not all the obligations expressed in the practice of human rights treaty bodies are reflected nationally. In particular, obligations 3, 5–14 are either not fully established in domestic law, addressed *ad hoc* during the crisis, not addressed in general, or their substance does not coincide with the substance of the requirements of the human rights treaty bodies.

The interview study allowed for advancing reflections on whether international human rights obligations reach the individual concerned in a specific case. The study illustrates that vaccination barriers exist in the areas where authorities have not worked on and those they tried to work with (see duties 5, 8, 9, 11, 12, 14, and 17, marked as asterisks in Table 1). The efforts to make vaccination economically affordable by providing free vaccination did not suffice for those whose income was lower than the price of tickets to the vaccination centres. Information concerning the free-of-charge vaccines was not necessarily perceived to be accurate because authorities informed migrants that they were entitled to ‘care that cannot wait’ only. Information that probably has been distributed was either not received or quickly forgotten. The information was not perceived to be relevant or required immediate actions due to recipients’ stress or lifestyle. Here, it is also interesting to observe that legal constructs are often perceived as a barrier to vaccination. Among those are limited access to care, so-called ‘care that cannot wait’, the absence of resident status, and electronic bookings and vaccination passports. Thus, the interview study can illustrate that such legislative readiness may not be sufficient to address the gaps and public health concerns, and more active work in identifying and addressing the gaps can be necessary.

This study, particularly its empirical part, can serve as an example of the issues raised during crises. Different groups of migrants, the same group in another country, or the group at different periods of time may have other barriers to vaccination. The critical message that international human rights sent is that measures should be tailored to the specific needs of each group. This means that authorities should strive to fulfil the obligations in Table 1. Obligations 7, to identify the gaps in immunization, and 8, to address the problems and gaps through tailored and targeted measures, indicate that there is no ‘one-size-fits all’ solution in public health crises. The barriers to vaccination and responses can and should be different. Lack of transparency in obligations and accountability for actions can result in the legal system’s unpreparedness to respond to subsequent crises.

The combination of the international law requirements with the national law example in the study allows reflecting that national administrative law is indispensable for the realization of human rights law (and vice versa). The difficulties in transposing international human rights obligation into national law can indicate a further need to specify the substance of the state’s obligations by international treaty bodies in accessible form for the states. These obligations here may be perceived as too abstract and not particularly specific. The difficulties of tracing the substance of the obligation related to the right to health regarding vaccination may be one reason for the problem with crisis preparedness. The interview study, particularly related to economic affordability (showing that offering vaccination free of charge does not make it affordable to everyone due to, in particular, transport costs), similarly calls for the international treaty bodies to further specify and reflect on the substance of obligations.

## Footnotes

1 World Health Organization, *Ten Threats to Global Health in 2019* (WHO 2024) <https://www.who.int/news-room/spotlight/ten-threats-to-global-health-in-2019> accessed 16 March 2024.

2 UK Health Security Agency, *Interactive Map of Vaccinations: Coronavirus in the UK* (GOV.UK March 2024) <https://coronavirus.data.gov.uk/details/interactive-map/vaccinations> accessed 16 March 2024; Regeringen och Regeringskansliet [The Government and the Government Office], *Skillnader i äldres vaccination mot COVID-19 ska kartläggas* [*Differences in the elderly’s vaccination against COVID-19 must be mapped*] (Regeringskansliet 11 October 2023) <https://www.regeringen.se/pressmeddelanden/2023/10/skillnader-i-aldres-vaccination-mot-covid-19-ska-kartlaggas/> accessed 16 November 2023.

3 Ted Dolby and others, ‘Monitoring Sociodemographic Inequality in COVID-19 Vaccination Uptake in England: A National Linked Data Study’ (2022) 76 JECH 646, 648; Long H Nguyen and others, ‘Self-reported COVID-19 Vaccine Hesitancy and Uptake Among Participants from Different Racial and Ethnic Groups in the United States and United Kingdom’ (2022) 13 Nature Communication 1, 2–3; Sarah Heiniger and others, ‘Differences in COVID-19 Vaccination Uptake in the first 12 months of Vaccine Availability in Switzerland—a Prospective Cohort Study’ (2023) 152 Swiss Medical Weekly 1.

4 OWID, *Data on COVID-19 (coronavirus) Vaccinations* (OWID 2023) <https://github.com/owid/covid-19-data/tree/master/public/data/vaccinations> accessed 19 December 2023; Ukrinform, *Nearly 45% of Adult Population in Ukraine Fully Jabbed against COVID-19* (Ukrinform 19 December 2023) <https://www.ukrinform.net/rubric-society/3381435-nearly-45-of-adult-population-in-ukraine-fully-jabbed-against-covid19.html> accessed 19 December 2023.

5 David Archard, Joe Brierley, Emma Cave, ‘Compulsory Childhood Vaccination: Human Rights, Solidarity, and Best Interests’ (2021) 29 Medical Law Review 716; Rogier Simons, Corrette Ploem and Johan Legemaate, ‘The Compatibility of Mandatory Vaccination with the European Convention on Human Rights: Implications for a National Vaccination Policy’ (2024) 31 European Journal of Health Law 285.

6 Committee on Economic, Social and Cultural Rights [CESCR], Concluding Observations: Greece, U.N. Doc E/C.12/1/Add.97 (7 June 2004) para 49; Concluding observations: Colombia, U.N. Doc E/C.12/1/Add.74 (6 December 2001) paras 25, 48; Concluding observations: Ukraine, U.N. Doc E/C.12/UKR/CO/6 (13 June 2014) para 19; Concluding observations: New Zealand, U.N. Doc E/C.12/NZL/CO/3 (31 May 2012) para 6; Concluding Observations, Guinea, U.N. Doc E/C.12/1/Add.5 (28 May 1996) para 11; European Committee of Social Rights [ECSR], Conclusions XXI-2-Germany—art 11-3, XXI-2/def/DEU/11/3/EN (8 December 2017); Committee on the Rights of the Child [CRC], Concluding observations: Romania, U.N. Doc CRC/C/ROU/CO/5 (13 July 2017) para 34(f); Concluding Observations: The Islamic Federal Republic of the Comores, U.N. Doc CRC/C/15/Add.14 (23 October 2000) para 34; Concluding observations: Poland, U.N. Doc CRC/C/POL/CO/5-6 (6 December 2021) para 34(e).

7 Vaccination has also been considered related to exercising other rights, such as the right to non-discrimination and private life. See eg *Vavřička and Others v the Czech Republic* [GC], App nos 47621/13 and 5 others (ECHR, 8 April 2021) para 263; *Andreas Dafnis v Greece*, CCPR/C/135/D/3740/2020 (CCPR, 26 January 2023) para 7.9; CRC, Concluding observations: Romania, U.N. Doc CRC/C/ROU/CO/5 (13 July 2017) para 34(f); Human Rights Committee [HRC], Concluding observations: Georgia, U.N. Doc CCPR/C/GEO/CO/5 (13 September 2022) paras 21–22.

8 See eg CESCR, General Comment No 3 The Nature of States Parties’ Obligations, U.N. Doc E/1991/23 (14 December 1990) paras 1, 10; General Comment No 14 [GC 14]: The Right to the Highest Attainable Standard of Health (art 12), U.N. Doc E/C.12/2000/4 (11 August 2000) para 47; Katharine G Young, ‘The Minimum Core of Economic and Social Rights: A Concept in Search of Content’ (2008) 33 Yale Journal of International Law 113, 115.

9 CESCR, GC 14, paras 43–44.

10 CRC, Concluding observations: Senegal, U.N. Doc CRC/C/SEN/CO/2 (20 October 2006) para 45(b); Concluding observations: Togo, U.N. Doc CRC/C/15/Add.255 (31 March 2005) paras 50–51; Concluding observations: Slovakia, U.N. Doc CRC/C/SVK/CO/3-5 (20 July 2016) paras 38–39; see also CRC, Concluding observations: Guinea-Bissau, U.N. Doc CRC/C/GNB/CO/2-4 (8 July 2013) paras 52; *Médecins du Monde—International v France*, Collective Complaint No 67/2011 (ECSR, 11 September 2012) para 160.

11 ECSR, Statement of interpretation on the right to protection of health in times of pandemic (21 April 2020) para 5.

12 However, the reference on the vaccination and AAAQ framework has been provided in particular in the CESCR, Statement on universal and equitable access to vaccines for the coronavirus disease (COVID-19), U.N. Doc E/C.12/2020/2 (15 December 2020) para 4.

13 CESCR, GC 14 para 12(a); Concluding observations: Bosnia and Herzegovina, U.N. Doc E/C.12/BIH/CO/3 (11 November 2021) para 43; CRC, General comment No 15 (2013) on the right of the child to the enjoyment of the highest attainable standard of health (art 24), U.N. Doc CRC/C/GC/15 (17 April 2013) para 25; ECSR, Conclusions 2017—Serbia—art 11-1, 2017/def/SRB/11/1/EN (8 December 2017); Conclusions XIX-2—Latvia—art 11-1, XIX-2/def/LVA/11/1/EN (2 January 2010); *International Commission of Jurists (ICJ) and others v Greece*, Collective Complaint No 173/2018 (ECSR, 26 January 2021) paras 225–227.

14 ECSR, Conclusions 2017—Serbia—art 11-3, 2017/def/SRB/11/3/EN (8 December 2017); ECSR, Statement of interpretation on the right to protection of health in times of pandemic (21 April 2020) para 5; CRC, Concluding observations: Guinea Bissau, U.N. Doc CRC/C/15/Add.177 (13 June 2002) para 35; Concluding observations: Georgia, U.N. Doc CRC/C/15/Add.124 (28 June 2000) para 45.

15 CRC, Concluding observations: Burundi, U.N. Doc CRC/C/15/Add.133 (16 October 2000) paras 54–55; Concluding observations: Latvia, U.N. Doc CRC/C/15/Add.142 (21 February 2001) paras 35–36; Concluding observations: Guinea-Bissau, U.N. Doc CRC/C/GNB/CO/2-4 (8 July 2013) paras 52–53; Concluding observations: Mozambique, U.N. Doc CRC/C/MOZ/CO/3-4 (27 November 2019) para 33(c).

16 ECSR, Statement of interpretation on the right to protection of health in times of pandemic (21 April 2020) para 5.

17 CRC, Concluding observations: Burkina Faso, U.N. Doc CRC/C/15/Add.19 (15 April 1994) para 10.

18 CRC, GC 15 para 41; Concluding observations: Pakistan, U.N. Doc CRC/C/PAK/CO/3-4 (15 October 2009) paras 60–61.

19 CESCR, GC 14 para 12(b).

20 CRC, Concluding observations: Nigeria, U.N. Doc CRC/C/NGA/CO/3-4 (21 June 2010) paras 60–61; Concluding observations: Georgia, U.N. Doc CRC/C/15/Add.124 (28 June 2000) paras 44–45; Concluding observations: Benin, U.N. Doc CRC/C/BEN/CO/2 (20 October 2006) para 52; Concluding observations: Guinea-Bissau, U.N. Doc CRC/C/GNB/CO/2-4 (8 July 2013) para 53; CESCR, Concluding observations: Kenya, U.N. Doc E/C.12/KEN/CO/1 (1 December 2008) para 32.

21 CESCR, Statement on universal and equitable access to vaccines for the coronavirus disease (COVID-19), U.N. Doc E/C.12/2020/2 (15 December 2020) para 5; Concluding observations: Kenya, U.N. Doc E/C.12/KEN/CO/1 (1 December 2008) para 32; Concluding observations: The Islamic Republic of Iran, U.N. Doc E/C.12/IRN/CO/2 (10 June 2013) para 16.

22 CESCR, General Comment No 19: The right to social security (art 9 of the Covenant), U.N. Doc E/C.12/GC/19 (4 February 2008) para 59.

23 CRC, Concluding observations: Ukraine, U.N. Doc CRC/C/UKR/CO/3-4 (21 April 2011) para 55; Concluding observations: Democratic Republic of Congo, U.N. Doc CRC/C/COD/CO/2 (10 February 2009) para 54; see also CESCR, Concluding observations: Latvia, U.N. Doc E/C.12/LVA/CO/2 (30 March 2021) para 41(c); Concluding observations: Denmark, U.N. Doc E/C.12/DNK/CO/5 (6 June 2013) para 18; Statement on universal and equitable access to vaccines for the coronavirus disease (COVID-19), U.N. Doc E/C.12/2020/2 (15 December 2020) para 4.

24 CRC, Concluding observations: Malawi, U.N. Doc CRC/C/MWI/CO/3-5 (6 March 2007) para 33(a).

25 CRC, Concluding observations: New Zealand, U.N. Doc CRC/C/NZL/CO/3-4 (11 April 2011) paras 37–38; Concluding observations: Gabon, U.N. Doc CRC/C/15/Add.171 (3 April 2002) para 69; General Comment No 9 (2006): The rights of children with disabilities, U.N. Doc CRC/C/GC/9 (27 February 2007) para 53; General Comment No 7 (2005): Implementing Child Rights in Early Childhood, U.N. Doc CRC/C/GC/7/Rev.1 (20 September 2006) para 27; CRC, Concluding observations: Costa Rica, U.N. Doc CRC/C/15/Add.117 (24 February 2000) para 21; Concluding observations: Viet Nam, U.N. Doc CRC/C/VNM/CO/3-4 (22 August 2012) para 57; Committee on the Rights of Persons with Disabilities [CRPD], Concluding observations: Malawi, U.N. Doc CRPD/C/MWI/CO/1-2 (5 October 2023) para 24(c); see also Committee on the Protection of the Rights of All Migrant Workers and Members of Their Families, General Comment No 2 on the rights of migrant workers in an irregular situation and members of their families, U.N. Doc CMW/C/GC/2 (28 August 2013) para 72; Concluding observations: Rwanda, U.N. Doc CMW/C/RWA/CO/2 (21 October 2021) para 9.

26 ECSR, Statement of interpretation on the right to protection of health in times of pandemic (21 April 2020) para 5.

27 CRC, Concluding observations: Slovakia, U.N. Doc CRC/C/SVK/CO/2 (10 July 2007) para 50.

28
*Médecins du Monde—International v France, Collective Complaint* No 67/2011 (ECSR, 11 September 2012) para 163; see also similar reasoning in Committee on the Elimination of Racial Discrimination [CERD], Concluding observations: Brazil, U.N. Doc CERD/C/BRA/CO/18-20 (19 December 2022) para 17(a).

29
*Médecins du Monde—International v France*, ibid para 161.

30 ibid para 163.

31 CESCR, GC 14 para 12(c).

32 CRC, Concluding observations: Georgia, U.N. Doc CRC/C/15/Add.124 (28 June 2000) para 44.

33 CRC, Concluding observations: Guatemala, U.N. Doc CRC/C/15/Add.154 (9 July 2001) paras 40–41; Concluding observations: Turkey, U.N. Doc CRC/C/15/Add.152 (9 July 2001) paras 51–52; Concluding observations: Slovakia, U.N. Doc CRC/C/SVK/CO/3-5 (20 July 2016) para 39; CESCR, Concluding Observations: Kenya, U.N. Doc E/C.12/KEN/CO/1 (1 December 2008) para 32.

34 CRC, Concluding observations: Serbia, U.N. Doc CRC/C/SRB/CO/2-3 (7 March 2017) para 46(e).

35 CESCR, Concluding observations: Bosnia and Herzegovina, U.N. Doc E/C.12/BIH/CO/3 (11 November 2021) paras 44–45.

36 CRC, Concluding observations: Turkey, U.N. Doc CRC/C/15/Add.152 (9 July 2001) paras 51–52; Concluding observations: Jamaica, U.N. Doc CRC/C/15/Add.32 (15 February 1995) para 27; Concluding observations: Guinea-Bissau, U.N. Doc CRC/C/GNB/CO/2-4 (8 July 2013) para 53.

37 CRC, Concluding observations: Viet Nam, U.N. Doc CRC/C/VNM/CO/3-4 (22 August 2012) para 58; Concluding observations: Slovakia, U.N. Doc CRC/C/SVK/CO/2 (10 July 2007) para 50.

38 CESCR, GC 14 para 12(d); CRC General comment No 15 (2013) on the right of the child to the enjoyment of the highest attainable standard of health (art 24), U.N. Doc CRC/C/GC/15 (17 April 2013) para 25.

39 CRC, Concluding observations: Slovakia, U.N. Doc CRC/C/SVK/CO/2 (10 July 2007) paras 49–50.

40 CRC, Concluding observations: the Democratic Republic of the Congo, U.N. Doc CRC/C/COD/CO/3-5 (28 February 2017) para 35(e); Concluding observations: Mozambique, U.N. Doc CRC/C/MOZ/CO/3-4 (27 November 2019) para 33(c).

41 CRC, Concluding observations: Egypt, U.N. Doc CRC/C/EGY/CO/3-4 (15 July 2011) para 63.

42 Nytt Juridiskt Arkiv [NJA] [Supreme Court Reports] 1973 p 423 (Swed.); Statens offentliga utredningar [SOU] [Swedish Government Official Report] 2008:125 En reformerad grundlag [Reformed Constitutiuon], para 484 (Swed); Ove Bring, ‘Monism och dualism igår och idag’ in Rebecca Stern and Inger Österdahl (eds), *Folkrätten i Svenk rätt* (Liber 2012) 16, 24–29; see also Anna-Sara Lind, ‘Folkrätten i den svenska konstitutionen’ Rebecca Stern and Inger Österdahl (eds), *Folkrätten i Svenk rätt* (Liber 2012) 147, 148.

43 SOU 2016:19 Barnkonventionen blir svensk lag [Convention on the Rights of the Child become a Swedish law] paras 133, 284 and 352 (Swed.).

44 However, the case law emphasizes that positive obligations derived from international conventions, including the implemented ones, cannot lead to the obligation of authorities to apply coercive measures on a person. See eg NJA 2016 p 1157 (Swed.).

45 SOU 1975:75 Medborgerliga fri- och rättigheter [Citizens’ rights and freedoms] para 184 (Swed.); Anna-Sara Lind, ‘The Right to Health from a Constitutional Perspective—the Example of the Nordic Countries’ in Elisabeth Rynning and Mette Hartlev (eds), *Nordic Health Law in a European Context* (Brill | Nijhoff 2011) 67, 71–72.

46 Regeringsformen [RF] [Instrument of Government] 12:2 (Swed.).

47 RF 1:6; 7:3.

48 Dagens Medicin, *Ukrainska flyktingar ska snabbt få vaccin* (17 March 2022) <https://www.dagensmedicin.se/specialistomraden/infektion/ukrainska-flyktingar-ska-snabbt-fa-vaccin/> accessed 10 February 2024.

49 1 ch 7§ Smittskyddslag [Infection Disease Control Act] (Svensk författingssamling [SFS] 2004:168) (Swed.).

50 1–4 §§ Förordning (SFS 2021:248) med instruktion för Folkhälsomyndigheten [Ordinance with instruction to Public Health Agency] (Swed.).

51 ibid 18 §.

52 Folkhälsomyndighet [Public Health Agency of Sweden], *Om det nationella vaccinationsregistret* (Folkhelsomyndighet 30 June 2023) <https://www.folkhalsomyndigheten.se/smittskydd-beredskap/vaccinationer/nationella-vaccinationsregistret/om-vaccinationsregistret/> accessed 21 December 2023; SOU 2024:2 Ett samordnat Vaccinationsarbete [A coordinated Vaccination work], paras 114ff (Swed.). The information submitted to the register includes a Swedish personal or temporary coordination number. Not all migrants with temporary migration statuses can have either of these numbers. This is one of the factors that make it difficult to follow up on the group’s vaccination coverage and identify vaccination gaps among the migrant population.

53 Folkhälsomyndighet, *Nationell plan för vaccination mot COVID-19* (Folkhälsomyndighet January 2022) <https://www.folkhalsomyndigheten.se/contentassets/c1a68cd812fd4034ae4d19fb7687ac51/nationell-plan-vaccination-covid-19.pdf> accessed 19 December 2023.

54 Folkhälsomyndighet, *Beslutunderlag, Rekommendation gällande smittskyddsinsatser till personer som ankommer till Sverige från Ukraina* (Folkhälsomyndighet 2022), <https://www.folkhalsomyndigheten.se/contentassets/42c69cb906b84a779d7122716ede1a96/beslutsunderlag-rekommendation-smittskyddsinsatser-personer-fran-ukraina.pdf> accessed 19 December 2023.

55 See Folkhälsomyndighet, Вакцинація проти грипу та COVID-19 (Ukrainian) (Folkhälsomyndighet 2023) <https://www.folkhalsomyndigheten.se/contentassets/7113939ff5eb4a89bb4f87509bec87b3/vaccination-mot-influensa-och-covid-19-pa-ukrainska.pdf> accessed 19 December 2023; Folkhälsomyndighet, Вакцинация от гриппа и COVID-19 (Russian) (Folkhälsomyndighet 2023) <https://www.folkhalsomyndigheten.se/contentassets/7113939ff5eb4a89bb4f87509bec87b3/vaccination-mot-influensa-och-covid-19-pa-ryska.pdf> accessed 19 December 2023; Folkhälsomyndigheten, *Information med anledning av kriget i Ukraina* (Folkhälsomyndighet 8 March 2023) <https://www.folkhalsomyndigheten.se/livsvillkor-levnadsvanor/halsa-i-olika-grupper/migration-och-halsa/ukraina/> accessed 19 December 2023.

56 Folkhälsomyndigheten, *Frågor och svar om vaccination mot COVID-19* (Folkhälsomyndighet 2023) <https://www.folkhalsomyndigheten.se/smittskyddberedskap/utbrott/aktuella-utbrott/covid-19/vaccination-mot-covid-19/fragor-och-svar-om-vaccination-mot-covid-19/> accessed 17 December 2023.

57 See also Justitieombudsmannen [JO] [Parliamentary Ombudsmen]: decision Nos 3527-2021 and 3576-2021 (14 June 2022) (Swed.) that criticized a county council for following the recommendations and not providing alternative vaccines.

58 1-2 §§ Lag (SFS 1994:137) om mottagande av asylsökande m.fl. [Act on Reception of Asylum Seekers] (Swed.).

59 See 6 § Förordning (SFS 1994:361) om mottagande av asylsökande m.fl. [Ordinance on Reception of Asylum Seekers] (Swed.).

60 8 ch. 10f §.Utlänningsförordning (SFS 2006:97) [Asylum Ordinance] (Swed.).

61 Note that the electronic system for submission was not established immediately. Letter of the Migration Agency (response to the author’s request for public information) from 19 December 2023. The information on the website was available at least from August 2022, see Migrationsverket, *Arbete, skola och sjukvård* (Migrationsverket 26 September 2023) <https://www.migrationsverket.se/Privatpersoner/Skydd-enligt-massflyktsdirektivet/Arbete-skola-och-sjukvard.html [https://web.archive.org/web/20220812054123/www.migrationsverket.se/Privatpersoner/Skydd-enligt-massflyktsdirektivet/Arbete-skola-och-sjukvard.html.]> accessed 19 December 2023.

62 The paragraph reads: ‘In Sweden, anyone aged 12 years or over can be vaccinated against COVID-19. Elderly and particularly vulnerable people may need to get vaccinated several times. It is very important that you have the vaccination you are offered if you are not already fully vaccinated, so that we can stop the spread of the virus together. Vaccinated against COVID-19 is free of charge and voluntary.’ The information is also available in Ukrainian and Russian, see Migrationsverket, *Arbete, skola och sjukvård* (Migrationsverket 2023) <https://www.migrationsverket.se/Privatpersoner/Skydd-enligt-massflyktsdirektivet/Arbete-skola-och-sjukvard.html> accessed 19 December 2023.

63 The Swedish Migration Agency also informs that contact information was provided at the time of moving for those who reside in the housing provided by it and municipalities.

64 8 ch. 1 § Hälso- och sjukvårdslagen (SFS 2017:30) [HSL] [Health and Medical Services Act] (Swed.), note, however, that municipalities can have certain limited responsibilities for healthcare of elderly individuals in care homes and children living in institutional care.

65 4 § Folkbokföringslag (SFS 1991:481) [The Population Registration Act] (Swed.); see also Skatteverket [Swedish Tax Agency], *Information till dig som kommer till Sverige från Ukraina* (Skatteverket 2023) <https://skatteverket.se/privat/folkbokforing/flyttatillsverige/informationtilldigsomkommertillsverigefranukraina.4.1657ce2817f5a993c3a2b8.html> accessed 19 December 2023.

66 Lag (SFS 2008:344) om hälso- och sjukvård åt asylsökande m.fl. [Act on Health and Medical Services for Asylum Seekers] (Swed.). Migrant children with temporary statuses are supposed to receive the same care as Swedish nationals. In 2023, five county councils decided to provide the same care for migrants, as for the nationals. Region Sörmland [Sörmland county council], Samverkanswebben, *Asyl- och migranthälsan Sörmland* (Region Sörmland 2023) <https://samverkan.regionsormland.se/for-vardgivare/halsoval/asyl—och-migranthalsa/> accessed 19 December 2023; Region Västerbotten [Västerbotten county council], *Riktlinje. Vård till migranter, dokumentnummer 67034* (1177 Region Västerbotten 2023) <https://rvlitablobprod.blob.core.windows.net/rvlitablobprod/67034.pdf> accessed 19 December 2023; Västmanland county council, *Vård i Västmanland om du är asylsökande eller saknar tillstånd för att vistas i Sverige* (1177 Region Västmanland 2023) <https://www.1177.se/Vastmanland/sa-fungerar-varden/vard-om-du-kommer-fran-ett-annat-land/vard-i-vastmanland-om-du-ar-asylsokande-gomd-eller-tillstandslos/> accessed 19 December 2023; Gävleborg county council, Beslut. Svar på Motion—Rätt till vård på lika villkor, LS 2014/1058 (3 February 2016). Östergötland county council, *Vård i Östergötland om du är asylsökande, gömd eller tillståndslös* (1177 Region Östragötaland 15 March 2023) <https://www.1177.se/Ostergotland/sa-fungerar-varden/vard-om-du-kommer-fran-ett-annat-land/vard-i-ostergotland-om-du-ar-asylsokande-gomd-eller-tillstandslos/[https://web.archive.org/web/20230315072607/https://www.1177.se/Ostergotland/sa-fungerar-varden/vard-om-du-kommer-fran-ett-annat-land/vard-i-ostergotland-om-du-ar-asylsokande-gomd-eller-tillstandslos/]> accessed 19 December 2023.

67 Proposition [Prop.] [Government bill] 2007/08:105 lag om hälso- och sjukvård åt asylsökande m.fl. [Act on Health Care and Medical Services for asylum-seekers, etc] paras 20, 29 (Swed.); SOU 2003:89 EG-rätten och mottagande av asylsökande [*EC Law and Reception of Asylum-seekers*] para 89 (Swed.). Persons who bear socially dangerous diseases can also receive treatment to protect others from the risk of being infected, which is not the case with the vaccination against COVID-19. 4 ch 6 § Smittskyddslag (SFS 2004:168) [Infection Disease Control Act] (Swed.).

68 SKR, *Rekommendation om kostnadsfrihet av Vaccinering mot COVID-19* (SKR 8 December 2020) <https://skr.se/download/18.71b542201784abfbf7ab911/1616494269672/Rekommendation-om-kostnadsfrihet-av-vaccinering-mot-covid-19.pdf> accessed 19 December 2023. The county councils often decide to provide such vaccinations free of charge. Kronoberg county council, letter (19 December 2023).

69 JO decision No 3527-2021 (14 June 2022), JO 2022/23, para 126 (Swed.); prop. 1981/82:97 om hälso- och sjukvårdslag, m.m, [about the Health and Medical Care Act, etc] para 111 (Swed.). COVID-19 vaccines are currently not considered to be a part of the general vaccination programmes, which has some impact on the division of responsibilities between the state and local authorities. SOU 2023:73 para 97 (Swed.).

70 1 ch. 9 § Smittskyddslag (SFS 2004:168) (Swed.).

71 ibid.

72 ibid 2 ch 3 §.

73 The requests were sent on 18 December 2023.

74 Sörmland county council, letter (December 2023); Sörmland county councile, *Boka tid för vaccination mot influensa och COVID-19 i Sörmland* (1177 Region Sörmland 2023) <https://www.1177.se/Sormland/undersokning-behandling/vaccinationer/sa-vaccinerar-du-dig-i-sormland/> accessed 19 December 2023. See also Västra Götaland county council, *Vaccination mot COVID-19 av flyktingar från Ukraina* (Västra Götalandsregionen 2023) <https://www.vgregion.se/halsa-och-vard/vardgivarwebben/vardriktlinjer/ukraina/vacc> accessed 19 December 2023.

75 Such as Arabic, Somali, Persian, and Tigre, though Ukrainian and Russian were not among them. Jönköping country council, letter (19 December 2023); Gotland county council, letter (8 January 2024), Dalarna county council, letter (27 January 2023), Gävleborg county council, letter (9 January 2024); Västernorrland county council, letter (8 January 2024); Uppsala county council, letter (12 January 2024).

76 Blekinge country council, letter (19 December 2023).

77 Kronoberg county council, letter (19 and 21 December 2023); Dalarna county council, letter (27 December 2023); Västmanland county council, letter (21 December 2023).

78 See Folkhälsa och sjukvård i Jönköpings län [Public health and healthcare in Jönköping county], *Hälsoguider—de som verkligen gör skillnad* (Folkhälsa och sjukvård i Jönköpings län 2023) <https://folkhalsaochsjukvard.rjl.se/folkhalsa/folkhalsa/folkhalsa/halsoguider—de-som-verkligen-gor-skillnad/> accessed 19 December 2023; Folkhälsa och sjukvård I Jönköpings län, *Modersmålsbaserad hälsokommunikation* (Folkhälsa och sjukvård i Jönköpings län 2023) <https://folkhalsaochsjukvard.rjl.se/folkhalsa/folkhalsa/folkhalsa/modersmalsbaserad-halsokommunikation/> accessed 19 December 2023; Jönköping country council, letter (19 December 2023); Västra Götaland county council, letters (19–20 December 2023).

79 Örebro country council, letter (22 December 2023).

80 KPMG, *Oberoende utvärdering av Region Stockholms hantering av COVID-19 (dnr 2020-0384)* para 54 (KPMG 13 October 2021) <https://share.mediaflowpro.com/?FPCFT8Y5BT> accessed 19 December 2023; Norrbotten county council, letter (20 December 2023).

81 Norrbotten county council, letter (20 December 2023).

82 ibid.

83 Belinda Bennett and Terry Carney, ‘Law, Ethics and Pandemic Preparedness: The Importance of Cross‐jurisdictional and Cross‐cultural Perspectives’ (2010) 34 Australian & New Zealand Journal of Public Law 106, 108.

84 Scania Country Council, letters (19 December 2023 and 5 February 2024).

85 Noel T Brewer and others, ‘Increasing Vaccination: Putting Psychological Science Into Action’ (2017) 18 Psychological Science in the Public Interest: A Journal of the American Psychological Society 149.

86 See acknowledgements section.

87 In fact, they perceived that they were only entitled to emergency care.

88 The participants had different perceptions of the most appropriate medium for information exchange. The majority preferred to have written information or built-in subtitles in Ukrainian videos. The subtitles that should be selected were occasionally considered technically difficult to find.

